# O050: Antibiotics in 70 German intensive care units: risk factors for high overall consumption

**DOI:** 10.1186/2047-2994-2-S1-O50

**Published:** 2013-06-20

**Authors:** S Schneider, F Schwab, P Gastmeier, E Meyer

**Affiliations:** 1 Hygiene and Environmental Medicine, Charite, Berlin, Germany

## Introduction

Due to increasing burden of multidrug resistant organisms and the lack of new antimicrobial substances prudent use of antibiotics (AB) becomes more essential than ever before. Surveillance of antimicrobial use is an important component of AB stewardship.

## Objectives

To explore, whether there are certain risk patterns in intensive care units (ICUs) determining the total AB use.

## Methods

We analysed the AB usage of 70 German ICUs in the year 2011. The data were collected in a standardized way within the SARI (Surveillance of Antimicrobial Use and Antimicrobial Resistance in ICUs) system. AB use densities (AD) were calculated as daily defined doses (DDD) per 1000 patient days (pd) for all systemically applied substances. We performed a stepwise forward multivariable analysis for the total AB use as binary outcome (total AD <= or > 75. percentile). The following variables were included: hospital type (university, teaching, other), hospital size (<= or > 600 beds), ward type (interdisciplinary, surgical, medical ICU), ward size (<= or > 12 beds), number of used AB substances, number of used AB groups, ADs of AB treatment groups and fractions of most used AB groups.

## Results

The median number of AB substances was 28 (range 10-36), the median number of AB groups was 17 (range 9–20). Median total AB use was 1285 DDD/1000 pd (range 639-2393). The fraction of the most used AB group ranged from 13 to 41% of total AB use (median 23%). In the multivariable analysis the group ADs of third generation (3G) cephalosporines, macrolides and methicillin resistant staphylococcus aureus (MRSA) active AB, respectively, were significantly associated with the total AB use in the logistic regression model. Interestingly, none of the structural parameters showed a significant association with total AB use.

## Conclusion

Diversity of AB usage is great among German ICUs, but structural parameters do not seem to determine significantly the extent of total AB use. Rather, usage of certain AB groups (3G cephalosporines, macrolides and MRSA active substances) seems to drive the overall AB consumption. However, whether this is due to certain patient characteristics or at least partially to imprudent AB use cannot be answered by our calculations alone and should be further investigated by complementary methods.

## Disclosure of interest

None declared.

